# Cannabidiol, a non-psychoactive cannabinoid, leads to EGR2-dependent anergy in activated encephalitogenic T cells

**DOI:** 10.1186/s12974-015-0273-0

**Published:** 2015-03-15

**Authors:** Ewa Kozela, Ana Juknat, Nathali Kaushansky, Avraham Ben-Nun, Giovanni Coppola, Zvi Vogel

**Affiliations:** The Dr Miriam and Sheldon G. Adelson Center for the Biology of Addictive Diseases, Sackler Faculty of Medicine, Tel Aviv University, Tel Aviv, Israel; Neurobiology Department, Weizmann Institute of Science, Rehovot, Israel; Immunology Department, Weizmann Institute of Science, Rehovot, Israel; Neurology Department, UCLA, Los Angeles, CA USA

**Keywords:** Cannabidiol, Memory T cells, LAG3, CD69, EGR2, T cell anergy

## Abstract

**Background:**

Cannabidiol (CBD), the main non-psychoactive cannabinoid, has been previously shown by us to ameliorate clinical symptoms and to decrease inflammation in myelin oligodendrocyte glycoprotein (MOG)35-55-induced mouse experimental autoimmune encephalomyelitis model of multiple sclerosis as well as to decrease MOG35-55-induced T cell proliferation and IL-17 secretion. However, the mechanisms of CBD anti-inflammatory activities are unclear.

**Methods:**

Here we analyzed the effects of CBD on splenocytes (source of accessory T cells and antigen presenting cells (APC)) co-cultured with MOG35-55-specific T cells (T_MOG_) and stimulated with MOG35-55. Using flow cytometry, we evaluated the expression of surface activation markers and inhibitory molecules on T cells and B cells. T_MOG_ cells were purified using CD4 positive microbead selection and submitted for quantitative PCR and microarray of mRNA transcript analyzes. Cell signaling studies in purified T_MOG_ were carried out using immunoblotting.

**Results:**

We found that CBD leads to upregulation of CD69 and lymphocyte-activation gene 3 (LAG3) regulatory molecules on CD4^+^CD25^−^ accessory T cells. This subtype of CD4^+^CD25^−^CD69^+^LAG3^+^ T cells has been recognized as induced regulatory phenotype promoting anergy in activated T cells. Indeed, we observed that CBD treatment results in upregulation of EGR2 (a key T cell anergy inducer) mRNA transcription in stimulated T_MOG_ cells. This was accompanied by elevated levels of anergy promoting genes such as IL-10 (anti-inflammatory cytokine), STAT5 (regulatory factor), and LAG3 mRNAs, as well as of several enhancers of cell cycle arrest (such as Nfatc1, Casp4, Cdkn1a, and Icos). Moreover, CBD exposure leads to a decrease in STAT3 and to an increase in STAT5 phosphorylation in T_MOG_ cells, positive and negative regulators of Th17 activity, respectively. In parallel, we observed decreased levels of major histocompatibility complex class II (MHCII), CD25, and CD69 on CD19^+^ B cells following CBD treatment, showing diminished antigen presenting capabilities of B cells and reduction in their pro-inflammatory functions.

**Conclusions:**

Our data suggests that CBD exerts its immunoregulatory effects *via* induction of CD4^+^CD25^−^CD69^+^LAG3^+^ cells in MOG35-55-activated APC/T_MOG_ co-cultures. This is accompanied by EGR2-dependent anergy of stimulated T_MOG_ cells as well as a switch in their intracellular STAT3/STAT5 activation balance leading to the previously observed decrease in Th17 activity.

## Background

Cannabinoids, the active materials found in *Cannabis* preparations (for example, in marijuana), have been shown to exert potent immunomodulatory and anti-inflammatory activities in various animal models of diseases with inflammatory background, including rheumatoid arthritis, experimental colitis, liver inflammation, brain injury, neurodegeneration, and multiple sclerosis (MS) (reviewed by [1,2]). MS is a neurodegenerative inflammatory disease of unknown trigger and complex neuroimmune pathology that involves myelin degeneration and CNS dysfunction. Encephalitogenic T cells specific for myelin components (primed by antigen presenting cells (APC)) have a key role in MS pathology [3,4] as well as in the mouse experimental autoimmune encephalomyelitis (EAE) model of MS [5]. We and others have shown that several cannabinoids including the main psychoactive Δ-9-tetrahydrocannabinol (THC) [6,7] and the main non-psychoactive cannabinoid, cannabidiol (CBD) [8] ameliorate CNS neuroinflammation and demyelination in EAE. Moreover, we have shown recently that CBD and THC decrease the myelin oligodendrocyte glycoprotein (MOG)35-55-induced T cell proliferation as well as the secretion of IL-17 and IL-6 cytokines [9], the key autoimmune cytokines that define the Th17 pathogenic phenotype [10,11]. Moreover, CBD increases the production of the anti-inflammatory IL-10 cytokine in these MOG35-55-stimulated T cells [9].

T cell effector functions and tolerance are controlled through multiple signaling pathways regulated by interactions with APC (and other accessory immune cells) and their surface molecules. Among the molecules shown to regulate memory T cell function, lymphocyte-activation gene 3 (LAG3; CD223) and CD69 have gained a major interest. LAG3 is a CD4 homolog that by interfering with major histocompatibility complex class II (MHCII) on APC upon antigen exposure [12] inhibits the function and expansion of memory T cells [13-15]. Furthermore, LAG3 upregulation induces early growth response 2 (EGR2)-dependent anergy (exhaustion) of activated T cells, this way limiting their pathogenic activity [16,17]. CD69 is a very potent inhibitory co-receptor that was found to serve as a constitutive suppressor of Th17 differentiation [18,19]. LAG3 and CD69 were reported to be induced on certain populations of CD4^+^CD25^−^ T cells [20,21] but were scarcely observed on the cell surface of CD4^+^CD25^+^ cells that serve as naturally occurring regulatory T cells (nTreg) [22]. Indeed, CD4^+^CD25^−^ T cells have been recently characterized as the main source of inducible non-conventional regulatory T cells [23,24] exerting their suppressive activity *via* a number of suppressory molecules including LAG3, CD69, IL-10, and TGFβ, and by this way promoting exhaustion of pathogenic T cells, mainly through EGR2-driven mechanisms [19,21,24,25].

There is almost no data describing the role of regulatory cell phenotypes and/or inhibitory co-receptors in the anti-inflammatory effects of cannabinoids. Therefore, we addressed this question using an *in vitro* system that employs interaction of encephalitogenic, MOG35-55 specific T cells (T_MOG_) with peripheral spleen-derived APC and naïve accessory T cells.

Antigen presentation to memory/encephalitogenic T cells is known to lead to activation of several cell cycle and effector pathways including the phosphatidylinositol-3-kinase/Akt/mTOR pathway, the mitogen-activated protein kinase (MAPK) pathway, and the Janus kinase/Signal transducers and Activators of Transcription (JAK/STATs) pathway [26,27]. Although Akt and MAPK pathways have been shown to be targeted by cannabinoids in various immune and non-immune cells [2,28], there is almost no data regarding the effect of cannabinoids on the activity of these pathways in inflammatory and autoimmune conditions. In this regard, we have recently shown that CBD exerts its anti-inflammatory activity in activated microglial cells *via* regulation of STAT1/STAT3 balance [29,30] demonstrating that STAT family members are targeted by CBD. STAT3 has been described as a key positive regulator of Th17 proliferation and function, including upstream regulation of RORγt-dependent production of IL-17 [31]. STAT5 is a major immunoregulatory factor restraining STAT3 pro-Th17 activity [32]. The ratio between STAT3 and STAT5 has been proposed to serve as a key factor determining the final pathogenic activity of autoreactive Th17 cells and its anergic propensity [33]. Moreover, EGR2 has been shown to act as an essential negative STAT3 regulator including IL-17 expression and Th17 expansion [34].

Herein, using an *in vitro* model of stimulated T_MOG_ cells co-cultured with spleen-derived CD19^+^ B cells serving as APC and other accessory CD4^+^ cells, we investigated the pathways and molecules mediating the immunoregulatory effects of CBD. The results show that CBD exerts its immunoregulatory effects *via* strong upregulation of CD69 and of LAG3 inhibitory molecules on CD4^+^CD25^−^ T cells. This is accompanied by EGR2, LAG3, and IL-10-dependent anergy of stimulated autoreactive T_MOG_ cells, followed by a shift in STAT3/STAT5 activation ratio leading to decreased Th17-like activity of CBD-treated memory encephalitogenic T cells observed by us previously.

## Materials and methods

### Reagents

Lyophilized MOG35-55 peptide [MEVGWYRSPFSRVVHLYRNGK] purchased from *GenScript* (Piscataway, NJ, USA) was reconstituted in sterile PBS and the stock solution stored in aliquots at −20°C. CBD was obtained from the National Institute on Drug Abuse (Baltimore, MD, USA) and was dissolved in ethanol. The dose 5 μM of CBD used here was selected based on our previous studies in which we showed that CBD at 5 μM significantly inhibited MOG-35-55-induced T_MOG_ cell proliferation and their Th17-like activity, that is, IL-17 release [8,9]. The final concentrations of ethanol in the various experiments did not exceed 0.1% and had no effect on the results. Fetal calf serum (FCS) and other tissue culture reagents were obtained from *Biological Industries* (Kibbutz Beit HaEmek, Israel).

### Encephalitogenic T cell line

The MOG35-55-specific T cell line (T_MOG_) was established from lymph node cells of C57BL/6 female mice that had been primed 10 days earlier with MOG35-55 emulsified in Complete Freund Adjuvant as previously described [5,8]. This T_MOG_ cell line has been maintained *in vitro* in RPMI-1640 containing 5% FCS and supplemented with recombinant human 10 U/ml of IL-2 (human/mouse cross-reactive, *Peprotech Inc*, Rocky Hill, NJ, USA; IL-2 enables T_MOG_ growth and expansion in between antigen stimulations), 2 mM L-glutamine, 100 μg/ml streptomycin, 100 U/ml penicillin, 50 μM β-mercaptoethanol, non-essential amino acids, and 1 mM sodium pyruvate (maintenance medium) with alternate stimulation with MOG35-55 (5 μg/ml) every 14 days as previously described [5,8].

### Flow cytometry analysis of immune cell phenotypes

T_MOG_ cells (1 × 10^6^ cells) were suspended in 4 ml of RPMI-1640 containing 2.5% FCS, 100 μg/ml streptomycin, 100 U/ml penicillin, 2 mM L-glutamine, and 50 μM β-mercaptoethanol (assay medium) and transferred to a 10-cm tissue culture dish. APCs (20 × 10^6^ cells) freshly isolated from spleens of 8-week naïve male C57BL/6 mice were added, and the mixture (APC/T_MOG_ co-cultures) was stimulated with 5 μg/ml of MOG35-55. Non-stimulated and MOG35-55-stimulated APC/T_MOG_ co-cultures were incubated in 37°C in a 5% CO_2_ incubator with or without CBD at 5 μM for 18 h. Then, the cells were spun down (5 min, 800 × *g*), the cell pellet resuspended in FACS buffer (PBS w/o Ca^++^/Mg^++^, 1% BSA), incubated with anti-mouse CD16/CD32 mAb (clone 93; *Biolegend*, San Diego, CA, USA), and aliquots of 1 × 10^6^ cells in 0.1 ml were subjected to staining at 4°C for 30 min using predetermined optimal concentrations of fluorophore-conjugated antibodies (all from *Biolegend*) including: CD4-FITC (H219.19), CD19-FITC (6D5), MHCII-PE (AF6-120.1), CD25-APC (3C7), CD69-PE (H1.2 F3), CD69-PE/Cy7 (H1.2 F3), and LAG3-PE (C9B7W). Staining was followed by two- or four-color immunofluorescence analysis by flow cytometry. Isotype- and concentration-matched control antibodies were used to assess non-specific staining. The cells were examined by BD LCSII flow cytometer (*Becton Dickinson*, Franklin Lakes, NJ, USA) using FACSDiva software (BD).

In additional control experiments, freshly isolated splenocytes were cultured without MOG35-55 (resting splenocytes), or T_MOG_ cells were cultured without APC and without MOG35-55 (resting T_MOG_ cells) for 18 h in the presence or absence of 5 μM CBD in maintenance medium. After this time, resting splenocytes or resting T_MOG_ cells were collected and processed by flow cytometry analysis as described above.

### CD4^+^ microbead purification of T_MOG_ cells from APC/T_MOG_ co-cultures

Dissociated spleen cells were plated in 10-cm plates (50 × 10^6^ cells/plate) in assay medium. After 2 h at 37°C in 5% CO_2_ humidified air to allow APC adherence, the media with non-adherent cells were removed, and the adherent APCs were gently washed with Ca^++^/Mg^++^ containing PBS and covered with a new assay medium. Then, 2.5 × 10^6^ of T_MOG_ cells were added and APC/T_MOG_ co-cultures were stimulated immediately with 5 μg/ml of MOG35-55 for 8 h in the presence or absence of CBD at 5 μM. CBD was added just prior to the addition of the MOG35-55. After 8 h of incubation, the media containing mostly T_MOG_ cells (but not the adherent APC cells) were carefully collected and spun down for 10 min at 800 × *g*. The cell pellet was washed in PBS containing 0.5% BSA and 2 mM EDTA, centrifuged again and resuspended in 90 μl of this buffer. To improve the purity of collected cells, CD4 (L3T4) magnetic beads (*Miltenyi Biotec GmbH,* Bergisch Gladbach, Germany) were added to the cell suspension for positive selection of CD4^+^ cells according to the manufacturer instructions. The CD4^+^ cells obtained this way (purified T_MOG_ cells) were subjected to protein phosphorylation assays (by immunoblotting) and to mRNA expression studies (by qPCR and gene arrays).

The incubation time of 8 h and the 5 μM dose of CBD were chosen based on previous time- and dose-response experiments, including cytokine production and release [9].

### CBD effects on signaling pathways

We checked the phosphorylation status of Akt, STAT3, and STAT5 using appropriate anti-phospho antibodies and Western blot analysis. The CD4 microbead purified T_MOG_ cells were rinsed twice with ice-cold PBS and lysed in RIPA buffer (140 mM NaCl, 20 mM Tris pH 7.4, 10% glycerol, 1% Triton X-100, 0.5% sodium deoxycholate, 0.1% sodium dodecyl sulfate, 2 mM EDTA) containing protease inhibitor cocktail (1:100, *Sigma*; St. Louis, MO, USA). Lysates were centrifuged at 4°C (10 min, 16,000 × *g*), pellets were discarded, and the supernatants were aliquoted and stored at −20°C for further analysis. Protein samples were subjected to 10% SDS-polyacrylamide gel electrophoresis as described earlier [29]. The blots were incubated overnight at 4°C with anti-phospho antibodies including: rabbit anti-p-Akt (Ser473; *Cell Signaling*, Danvers, MA, USA), rabbit anti-p-STAT3 (Tyr705; *Abcam*, Cambridge, UK), rabbit anti-p-STAT3 (Ser727; *Cell Signaling*), rabbit anti-p-STAT5 (Tyr694; *Cell Signaling*), as well as mouse anti-total STAT3 protein (*Cell Signaling*). Horseradish peroxidase (HRP)-conjugated secondary goat anti-rabbit or anti-mouse antibodies (*Jackson ImmunoResearch Laboratories*, Inc., West Grove, PA, USA) were applied for 2 h at room temperature and the blots visualized using the enhanced chemiluminescence detection kit (EZ-ECL, *Biological Industries,* Kibbutz Beit Haemek, Israel). The blots were scanned and quantified with NIH Image 1.63. Levels of β-actin and the total STAT3 proteins were used as loading controls and for further data normalization.

We would like to note that 8 h MOG35-55 stimulation resulted in a lower yield of encephalitogenic T cells following the use of the CD4 microbead system. We assume that this change in CD4 yield reflects transient downregulation (internalization and turnover) in CD4 receptor expression following antigen stimulation [35,36]. Indeed, immunoblotting of STAT3 and β-actin proteins confirmed a decrease in total level of both of these proteins by about 35% in all MOG-treated samples. This observation corresponds well with these previous reports on post-stimulation CD4 receptor downregulation [35,36]. Addition of 5 μM CBD had no further effect.

### RNA extraction, quantitative PCR (qPCR) and microarray transcript analysis

The *Miltenyi* CD4 bead purified T_MOG_ cells were lysed and subjected to RNA extraction (*5Prime*, Darmstadt, Germany) followed by qPCR analysis as reported earlier [9]. The cDNA of each specific gene was amplified using a pair of specific primers as detailed in Table 1. Quantification was performed by ‘the comparative cycle of threshold method’ using β2-microglobulin (β2MG) gene product for normalization [30]. The qPCR runnings were repeated 3 to 4 times using mRNA preparations from independent experiments.

**Table 1:** **Primers used for qPCR analysis of mRNA levels in purified T**_**MOG**_ **cells co-cultured previously with adherent APCs**

**Gene**	**Accession number**	**Forward**	**Reverse**
β_2_MG	NM_009735	AGTTCCACCCGCCTCACATTGAAA	TCGGCCATACTGGCATGCTTAACT
STAT5	NM_010118.3	AGTTGTGGAGAGAGAAACAATC	ACACCATAGTCAATAAGCCATC
LAG3	NM_008479.2	CTGCCTTAGAACATGGGATTC	CCATCTCCGTCTCCAGTT
EGR2	NM_010118.3	AGT TGTGGAGAGAGAAACAAT C	ACACCATAGTCAATAAGCCATC
IL-10	NM_010548.2	CCTTTGCTATGGTGTCCTTTC	GGATCTCCCTGGTTTCTCTT

For microarray transcripts, 200 ng samples of total RNA were amplified, labeled and hybridized onto *Illumina* MouseRef-8 v 2.0 Expression Bead-Chip (*Illumina* Inc., San Diego, CA, USA), querying the expression of >24,000 RefSeq-curated gene targets and 796 random sequences (used for the assessment of background noise). Arrays were processed and scanned with *Illumina* BeadStation platform according to the manufacturer’s protocol. Raw data were analyzed using the Bioconductor packages (http://www.bioconductor.org; [37]).

### Statistical analysis

Data is expressed as the mean ± SEM of two to four independent experiments and analyzed for statistical significance using one way analysis of variance (ANOVA), followed by Newman-Keul’s or Dunnett’s *post hoc* tests. *P* < 0.05 was considered significant. *Graph Pad Prism* program (La Jolla, CA, USA) was used for statistical analysis of the data.

### Study approval

All mice used in the studies were maintained according to the guidelines of the Institutional Animal Care and Use Committee (IACUC). Animal experiments were approved and performed by the Weizmann Institute of Science and the Tel Aviv University IACUCs.

## Results

### CD4^+^CD25^+^ cell number is decreased by MOG35-55-stimulation but not affected by CBD treatment

**Figure 1:**
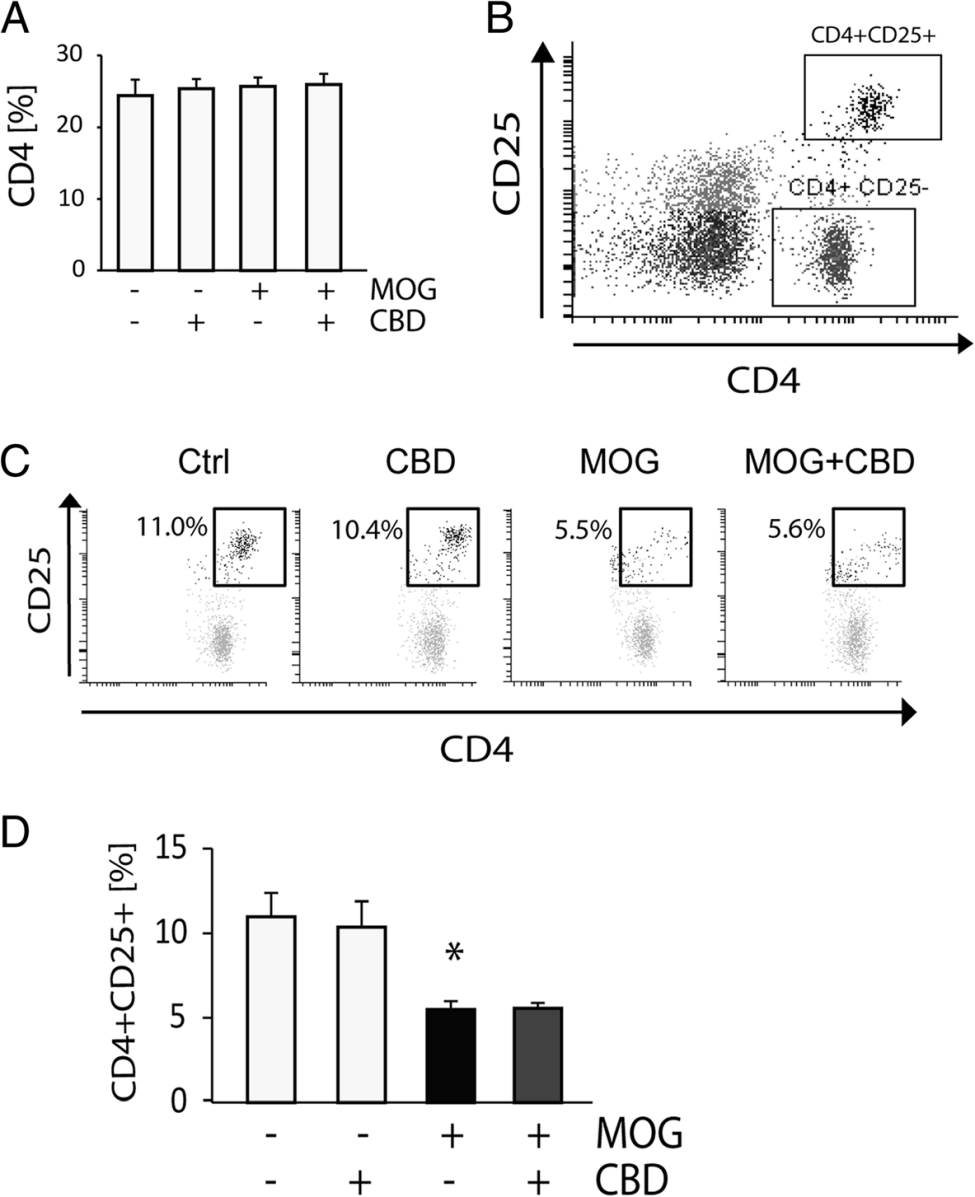
**Decrease in CD4** ^**+**^ **CD25** ^**+**^ **cells in MOG35-55-stimulated APC/T**_**MOG**_ **co-cultures is not affected by CBD.** Splenocytes (including accessory CD4^+^ T cells) were co-cultured with T_MOG_ cells and stimulated with MOG35-55 at 5 μg/ml. CBD at 5 μM was added to the cells immediately before MOG35-55. **(A)** Bar graph showing the average percentage of CD4^+^ cells in APC/T_MOG_ co-cultures (100% as equal to total number of cells). Neither treatment affected overall CD4^+^ T cell number; **(B)** dot plot histogram presenting CD4^+^ T cells in APC/T_MOG_ co-cultures positive and negative for CD25 expression; **(C)** a representative dot plot showing the changes in CD4^+^CD25^+^ T cell population in control or MOG35-55-treated APC/T_MOG_ co-cultures in the presence or absence of CBD; **(D)** bar graph showing the number of cells positive for CD4 and CD25 ± SEM. ANOVA *F*(3,8) = 7.8, *P* < 0.01. *P < 0.05 *vs* non-stimulated cells. *n* = 3 to 4.

T_MOG_ cells were co-cultured with splenocytes (APC/T_MOG_ co-cultures) and stimulated with MOG35-55 at 5 μg/ml for 18 h in the presence or absence of 5 μM CBD, and the number of CD4^+^ cells was determined using flow cytometry. As presented in Figure 1A, neither MOG35-55-stimulation nor CBD addition affected the total number of CD4^+^ cells in these co-cultures. Next, we analyzed the frequency of CD4^+^CD25^+^ natural regulatory T cells in APC/T_MOG_ co-cultures (Figure 1B). We observed that in control, non-stimulated co-cultures 11.0% ± 1.4% of CD4^+^ cells were CD25 positive (CD4^+^CD25^+^) and this frequency did not change following CBD treatment (10.4% ± 1.5%; Figure 1C,D). MOG35-55-stimulation of these APC/T_MOG_ co-cultures led to a significant decrease in CD4^+^CD25^+^ cells (down to 5.5% ± 0.5% of all CD4^+^, *P* < 0.05), and this level was not affected by CBD co-incubation (5.6% ± 0.3%).

### CBD induces upregulation of CD4^+^CD25^−^CD69^+^LAG3^+^ cells in MOG35-55-stimulated APC/T_MOG_ co-cultures

**Figure 2:**
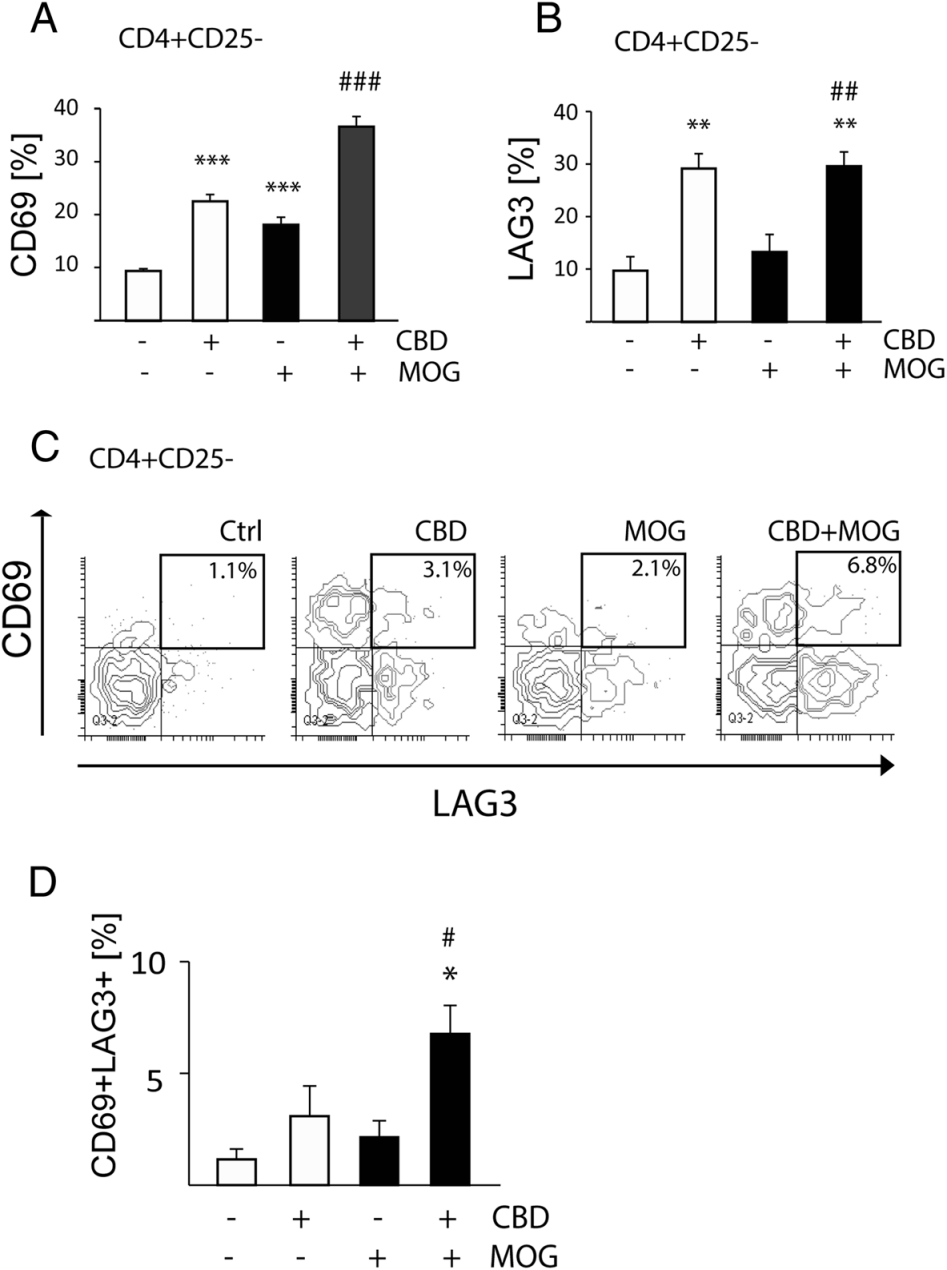
**CBD treatment results in upregulation of CD69 and of LAG3 regulatory molecules on CD4** ^**+**^ **CD25** ^**−**^ **T cells in MOG35-55-stimulated APC/T**_**MOG**_ **co-cultures.** APC/T_MOG_ co-cultures were treated with MOG35-55 and CBD. **(A)** Percentage of CD4^+^CD25^−^ T cells expressing CD69 (ANOVA *F*(3,12) = 68.9; *P* < 0.001); **(B)** percentage of CD4^+^CD25^−^ T cells expressing LAG3 (ANOVA *F*(3,12) = 13.0, *P* < 0.001); **(C)** the representative contour plot density graphs showing the co-expression of CD69 and LAG3 in the CD4^+^CD25^−^ subpopulation; **(D)** percentage of CD4^+^CD25^−^ cells expressing both CD69 and LAG3 ± SEM. ANOVA *F*(3,7) = 7.3, *P* < 0.05. *P < 0.05, **P < 0.01, ***P < 0.001 *vs* non-stimulated cells; ^#^ *P* < 0.05, ^##^ *P* < 0.01, ^###^ *P* < 0.001 *vs* MOG35-55-stimulated cells. *n* = 3 to 4.

At the second step, we analyzed the CD4^+^CD25^−^ population for the expression of CD69 molecule. In control, non-stimulated APC/T_MOG_ co-cultures 9.3% ± 0.4% of the CD4^+^CD25^−^ cells were found to be positive for CD69 (Figure 2A). Interestingly, 18 h co-incubation with CBD resulted in a very significant increase in CD4^+^CD25^−^CD69^+^ cells reaching 22.5% ± 1.3% (*P* < 0.001 *vs* 9.3% ± 0.4% observed in the non-stimulated APC/T_MOG_ co-cultures). MOG35-55-stimulation of APC/T_MOG_ co-cultures resulted in doubling the CD69 expression on CD4^+^CD25^−^ cells up to 18.0% ± 1.5% (*P* < 0.001 *vs* non-stimulated cells), and CBD addition together with MOG35-55 further increased the frequency of CD4^+^CD25^−^CD69^+^ cells up to 36.6% ± 1.9% (*P* < 0.001 *vs* MOG-treated cells).

Following incubation with CBD, we observed increased expression of LAG3 regulatory molecule on CD4^+^CD25^−^ T cells. The basal number of CD4^+^CD25^−^ cells expressing LAG3 (9.7% ± 2.6%) was increased by approximately three times reaching 29.0% ± 2.8% in the presence of CBD (Figure 2B). MOG35-55 stimulation did not significantly affect the number of CD4^+^CD25^−^LAG3^+^ cells (13.3% ± 3.3%) as compared to non-stimulated APC/T_MOG_ co-cultures. Addition of CBD to MOG35-55-stimulated APC/T_MOG_ co-cultures resulted in a similar level of increase in LAG3 as observed in non-stimulated cells (reaching 29.6% ± 2.7% of the total CD4^+^CD25^−^ cells; Figure 2B).

Co-expression of CD69 and LAG3 molecules on CD4^+^CD25^−^ T cells has been defining an inducible non-classical regulatory T cell phenotype [25]. The number of CD4^+^CD25^−^ cells double positive for CD69 and LAG3 in control APC/T_MOG_ co-cultures was 1.1% ± 0.9% of the total CD4^+^CD25^−^ cells and this frequency was not significantly affected in the presence of CBD (3.1% ± 2.7%; Figure 2C,D). MOG35-55 stimulation of APC/T_MOG_ co-cultures did not significantly affect the number of CD4^+^CD25^−^CD69^+^LAG3^+^ cells (2.1% ± 1.5%). However, CBD treatment led to a three-fold increase in CD4^+^CD25^−^CD69^+^LAG3^+^ cells, reaching 6.8% ± 2.6% (*P* < 0.05) in MOG35-55-treated cells (Figure 2C,D).

As controls, we analyzed the effects of CBD treatment on the expression of CD69 and of LAG3 on resting splenocytes cultured without T_MOG_ and without MOG35-55 as well as in resting T_MOG_ cultured without MOG35-55 and without APC (both in maintenance medium). These control experiments showed that the levels of CD69 (6.9% ± 1.2%) and of LAG3 (0.5% ± 0.3%) in resting spleen-derived CD4^+^ splenocytes cultured separately were not significantly affected by CBD treatment (reaching 8.7% ± 1.7% and 0.4% ± 0.4%, respectively; Table 2). Similarly, in resting T_MOG_ cells cultured alone, CBD treatment did not affect the basal levels of CD69 (0.9% ± 0.3% in control cells and 1.0% ± 0.2% in CBD-treated) and of LAG3 (0.7% ± 0.2% and 0.5% ± 0.1%, respectively). These experiments demonstrate that the changes in CD69 and LAG3 expression are induced by CBD treatment only in APC/T_MOG_ co-cultures and not in T_MOG_ or in splenocytes cultured separately.

**Table 2:** **CBD treatment does not affect CD69, CD25, and LAG3 expression in resting cells**

**Phenotype**	**Control (%)**	**CBD (%)**	***T*** **test**
Resting splenocytes			
CD4^+^	20.0 ± 2.7	20.7 ± 2.2	ns
CD4^+^CD69^+^	6.9 ± 1.2	8.7 ± 1.9	ns
CD4^+^LAG3^+^	0.5 ± 0.3	0.4 ± 0.4	ns
CD19^+^	59.4 ± 2.0	59.4 ± 2.1	ns
CD19^+^CD69^+^	13.6 ± 3.4	17.4 ± 3.4	ns
CD19^+^CD25^+^	2.5 ± 0.2	3.1 ± 0.2	ns
Resting TMOG cells			
CD69^+^	0.9 ± 0.3	1.0 ± 0.2	ns
LAG3^+^	0.7 ± 0.2	0.5 ± 0.1	ns

Freshly isolated mouse splenocytes (including accessory CD4^+^ T cells and CD19^+^ B cells) or resting T_MOG_ cells were maintained separately for 18 h in maintenance medium without MOG35-55 and in the presence or absence of CBD at 5 μM. CD69, LAG3 and CD25 surface expression was analyzed using flow cytometry (*n* = 3). Data are expressed as percentage ± SEM defined as the percent of cells expressing relevant antigen within an appropriate parent population.

ns, not significant.

### mRNA levels of anergy-associated genes are upregulated in MOG35-55-stimulated T_MOG_ cells and enhanced by CBD

CD4^+^CD25^−^ T cells expressing CD69 and/or LAG3 have been shown to be potent inducers of anergy in activated effector/memory T cells *via* upregulating EGR2 transcription factor [16,17]. Thus, we examined the levels of EGR2 mRNA in non-stimulated and in MOG35-55-stimulated T_MOG_ cells co-cultured with spleen-derived APC.

**Figure 3:**
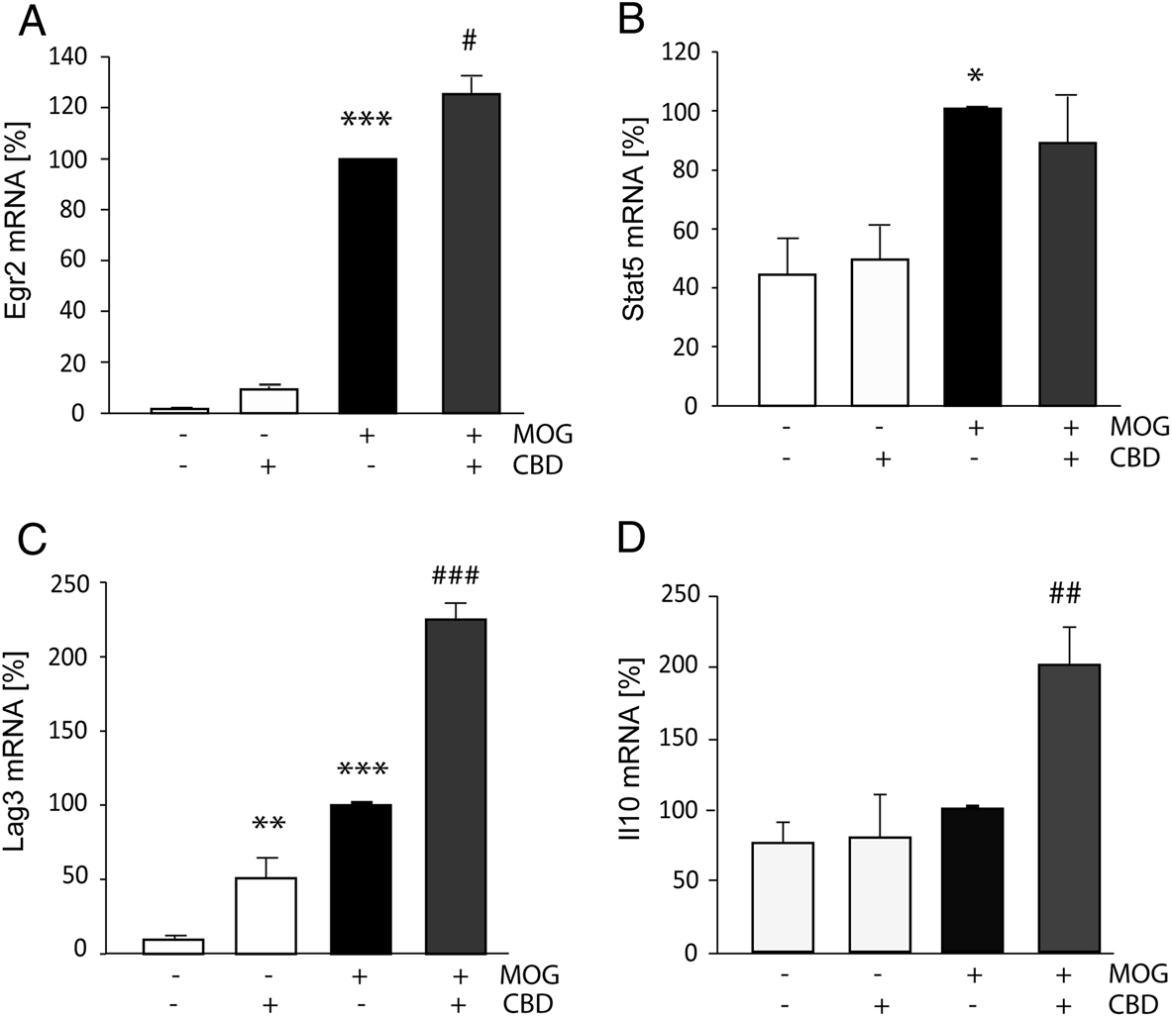
**The effect of CBD treatment on EGR2, STAT5, LAG3, and IL-10 mRNA levels in purified T**_**MOG**_ **previously co-cultured with APC.** T_MOG_ cells were co-cultured with adherent APC and stimulated with MOG35-55 in the presence or absence of CBD. T_MOG_ cells were then purified using CD4^+^ microbeads, lysed, and subjected for mRNA extraction and qPCR analysis using gene specific primers. The bar graphs show the levels of the indicated mRNAs as percentage of the amounts observed following stimulation with MOG35-55. **(A)** EGR2 mRNA (ANOVA *F*(3,4) = 211.5, *P* < 0.001); **(B)** STAT5 mRNA (ANOVA *F*(3,12) = 6.1, *P* < 0.01); **(C)** LAG3 mRNA (ANOVA *F*(3,8) = 116.5, *P* < 0.001); **(D)** IL-10 mRNA (ANOVA *F*(3,11) = 9.9, *P* < 0.01); (*n* = 2 to 4). Symbols: *P < 0.05, **P < 0.01, ***P < 0.001 *vs* non-stimulated cells; ^#^ *P* < 0.05, ^##^ *P* < 0.001, ^###^ *P* < 0.001 *vs* MOG35-55-stimulated cells.

T_MOG_ cells were activated with MOG35-55 for 8 h in the presence of adherent APC. The floating T_MOG_ cells were then collected and purified using CD4 microbeads and their mRNA subjected for qPCR analysis of EGR2 mRNA. We found that MOG35-55 stimulation dramatically upregulated the expression of EGR2 mRNA in the purified T_MOG_ cells as compared to control cells (*P* < 0.01; Figure 3A). This effect was potentiated by CBD treatment by another 25% (*P* < 0.05). CBD itself slightly but insignificantly increased the expression of EGR2 mRNA in non-stimulated T_MOG_ cells.

In parallel, we have evaluated the mRNA levels of other anergy promoters including STAT5 transcription factor, LAG3, and the IL-10 anti-inflammatory cytokine. Figure 3B shows that MOG35-55 stimulation increased STAT5 mRNA in T_MOG_ cells (by twofold; *P* < 0.05). CBD did not have any further effect on MOG35-55-upregulated STAT5 mRNA expression. CBD did not affect the basal STAT5 levels either (Figure 3B). Interestingly, CBD significantly upregulated (by *ca* fivefold) the level of LAG3 mRNA in control, non-stimulated T_MOG_ cells previously co-cultured with adherent APC (*P* < 0.01; Figure 3C). Stimulation with MOG35-55 led to a very high LAG3 mRNA upregulation (by *ca* tenfold, *P* < 0.001). Moreover, the amount of LAG3 mRNA expression was further increased in the presence of CBD by additional 120% (*P* < 0.001; Figure 3C). In the case of IL-10, neither CBD addition nor MOG35-55 stimulation affected the level of IL-10 mRNA expression (Figure 3D). However, CBD treatment of MOG35-55-stimulated cells resulted in a significant IL-10 mRNA upregulation of 100% above the level observed in MOG35-55-treated T_MOG_ cells (*P* < 0.01; Figure 3D).

We performed gene array analysis of mRNA expression in T_MOG_ cells in search for additional transcripts that are involved in anergy and tolerogenic processes. As shown in Table 3, MOG35-55-stimulation of T_MOG_ cells led to a significant upregulation of various anergy promoters representing the following categories: regulators of cell cycle (Ndrg1 by 6.2-fold, Cdkn1a by 2.6-fold, Casp4 by 2.2-fold, and Fas by 2.6-fold), tolerance inducers (Lag3 by 1.7-fold, Icos by 1.6-fold, Nfatc1 by 3.3-fold), and chemokine recruiting regulatory T cells (Ccl4 by 9.1-fold). The addition of CBD treatment significantly potentiated the MOG35-55-upregulated transcript levels of tolerance inducers, that is, of Lag3 (by 305%), Icos (by 43%), and Nfatc1 (by 21%) and of cell cycle regulators such as Cdkn1a (by 19%), Casp4 (by 22%), and Fas (by 27%). It did not affect the MOG35-55-enhanced levels of Ndrg1 and Ccl4. Treatment with CBD alone resulted in more than twofold increase of the levels of Icos, Ndrg1, and Casp4 in non-stimulated T_MOG_ cells (*P* < 0.005). Slight but significant increases were observed following CBD treatment for Lag3, Nfatc1, and Fas mRNA transcripts in non-stimulated T_MOG_ cells.

**Table 3:** **mRNA microarray analysis of CBD and MOG35-55 modulation of T**_**MOG**_ **cell activity**

**Gene**	**Full name**	**MOG (fold change)**	**MOG + CBD (fold change)**	**CBD (fold change)**
Lag3	Lymphocyte-activation gene 3	1.7	**5.2**	*1.7*
Icos	Inducible T-cell costimulator	1.6	**2.3**	*2.1*
Nfatc1	Nuclear factor of activated T-cells, cytoplasmic 1	3.3	**4.0**	*1.3*
Ndrg1	N-myc downstream-regulated gene 1	6.2	6.7	*2.5*
Cdkn1a	Cyclin-dependent kinase inhibitor 1A	2.6	**3.1**	1
Casp4 (Casp11)	Caspase 4, apoptosis-related cysteine peptidase	2.2	**2.7**	*2.1*
Fas	Fas cell surface death receptor	2.6	**3.3**	*1.3*
Ccl4	Chemokine (C-C motif) ligand 4	9.1	9	0.9

T_MOG_/adherent APC co-cultures were stimulated with MOG35-55 in the presence or absence of CBD. mRNA from CD4^+^ microbead purified T_MOG_ cells was subjected to microarray analysis. The table presents the genes significantly upregulated by MOG35-55 and their modulation by CBD (*P* < 0.005). The data are expressed as fold change *vs* purified T_MOG_ cells that were not stimulated by MOG35-55 when co-cultured with APC. Numbers in bold indicate significant effect of CBD *vs* the MOG35-55-induced effect. Values in italics indicate significant effects of CBD *vs* non-stimulated T_MOG_ cells (*P* < 0.005).

### Th17 signature signaling pathways are affected by CBD in T_MOG_ cells co-cultured previously with APC

EGR2 was previously reported to control STAT3 and STAT5 activities, the main respective positive and negative regulators of Th17 phenotype [33]. Therefore, we evaluated the levels of STAT3 and STAT5 activation in T_MOG_ cells. As described above, T_MOG_ cells co-cultured with adherent spleen-derived APCs were stimulated with MOG35-55 in the presence or absence of 5 μM CBD. After 8 h of stimulation, CD4 microbead purified T_MOG_ cells were lysed, subjected to gel electrophoresis, and immunostained for phospho-STAT3 (at Tyr705 and Ser727) and for phospho-STAT5 (at Tyr694).

**Figure 4:**
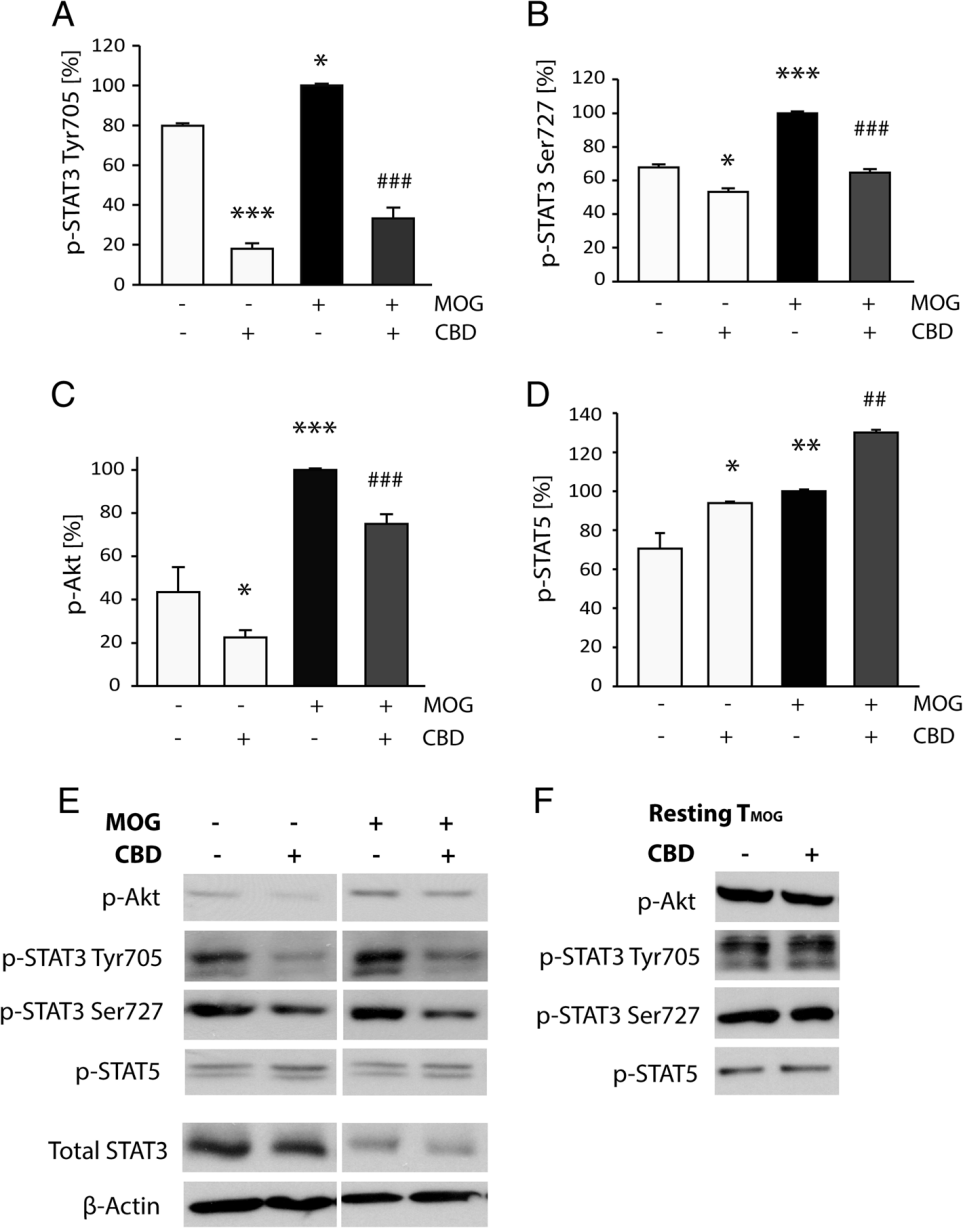
**CBD modulates Th17 signaling pathways in T**_**MOG**_ **cells co-cultured with spleen-derived APC.** Pre-adhered APC were co-cultured with T_MOG_ cells and stimulated with MOG35-55 in the presence or absence of CBD. CD4^+^ microbead purified T_MOG_ cells were lysed and proteins subjected to gel electrophoresis, followed by transfer to nitrocellulose membrane and immunostaining of the phosphorylated forms of Akt, STAT3, and STAT5 as well as of total amount of β-actin and STAT3. The bar graphs show average percentage values (in comparison to MOG35-55 without CBD as 100%) of **(A)** phospho-STAT3 Tyr705 (ANOVA *F*(3,4) = 94.3, *P* < 0.001); **(B)** phospho-STAT3 Ser727 (ANOVA *F*(3,4) = 96.7, *P* < 0.001), **(C)** phospho-Akt (ANOVA *F*(3,4) = 96.7, *P* < 0.001); **(D)** phospho-STAT5 (ANOVA *F*(3,7) = 32.6, *P* < 0.001); **(E)** representative blots showing the phosphorylated forms of Akt, STAT3 (Tyr705 and Ser727), and STAT5 Tyr694 as well as total levels of β-actin and STAT3 (*n* = 2 to 3); **(F)** in this control experiment, resting T_MOG_ cells were cultured without APC and without MOG35-55 for 8 h in the presence or absence of 5 μM CBD in maintenance medium. Representative immunoblots (*n* = 2) show that CBD does not affect the levels of phospho-Akt, phospho-STAT3, and phospho-STAT5 in resting T_MOG_ cells cultured without APC. Symbols: *P < 0.05, **P < 0.01, ***P < 0.001 *vs* non-stimulated purified T_MOG_; ^##^ *P* < 0.001, ^###^ *P* < 0.001 *vs* MOG35-55-treated T_MOG_ cells.

A relatively high amount of STAT3 phosphorylated at Tyr705 (a major activating residue) as well as at Ser727 (enhancing STAT3 dimerization, translocation to the nucleus and DNA binding) was observed in T_MOG_ cells even without stimulation with MOG35-55 (Figure 4A,B,E). MOG35-55 stimulation led to a small, but significant, increase in the levels of phospho-STAT3 at both Tyr705 (by 20%, *P* < 0.05) and Ser727 (by 32%, *P* < 0.001). CBD treatment led to a large decrease in Tyr705 phospho-STAT3 in both non-stimulated (reduction by 67%) and stimulated T_MOG_ cells (reduction by 68%; *P* < 0.001). CBD decreased slightly, but significantly (by 15%, *P* < 0.05), the phosphorylation of STAT3 at position Ser727 in non-stimulated T_MOG_ cells and had a larger effect (decrease of 35%, *P* < 0.001) on Ser727 phosphorylation in the presence of MOG35-55 (Figure 4B,E).

Interestingly, both CBD treatment and MOG35-55 stimulation increased STAT5 phosphorylation (at Tyr694). CBD addition to non-stimulated T_MOG_ cells resulted in increased phospho-STAT5 levels (by 24%, *P* < 0.05), MOG35-55 stimulation resulted in increased phospho-STAT5 levels of 30% (*P* < 0.01). Applying MOG35-55 and CBD together resulted in an additive effect of 60% (*P* < 0.01, Figure 4D,E).

The Akt pathway is known to positively regulate effector T cells’ proliferation while restraining intracellular regulatory mechanism [27]. Indeed, phospho-Akt levels were increased by 57% (*P* < 0.001) in stimulated T_MOG_ cells co-cultured previously with APC. CBD treatment decreased Akt phosphorylation by 25% (*P* < 0.001; Figure 4C,E). Moreover, CBD treatment also decreased phospho-Akt levels in non-stimulated T_MOG_ cells by 20% (*P* < 0.05).

We performed control experiments to evaluate if CBD treatment affects the activity of STAT3, STAT5, and Akt pathways in resting T_MOG_ cells cultured in maintenance medium without MOG35-55 stimulation and without splenocytes. Figure 4F shows that CBD did not affect the phospho-STAT3 (Tyr705, Ser727), phospho-Akt, and phospho-STAT5 levels in these resting T_MOG_ cells.

### The expression of MHCII, CD25, and CD69 on CD19^+^ B cells is downregulated following CBD treatment in MOG-stimulated APC/T_MOG_ co-cultures

**Figure 5:**
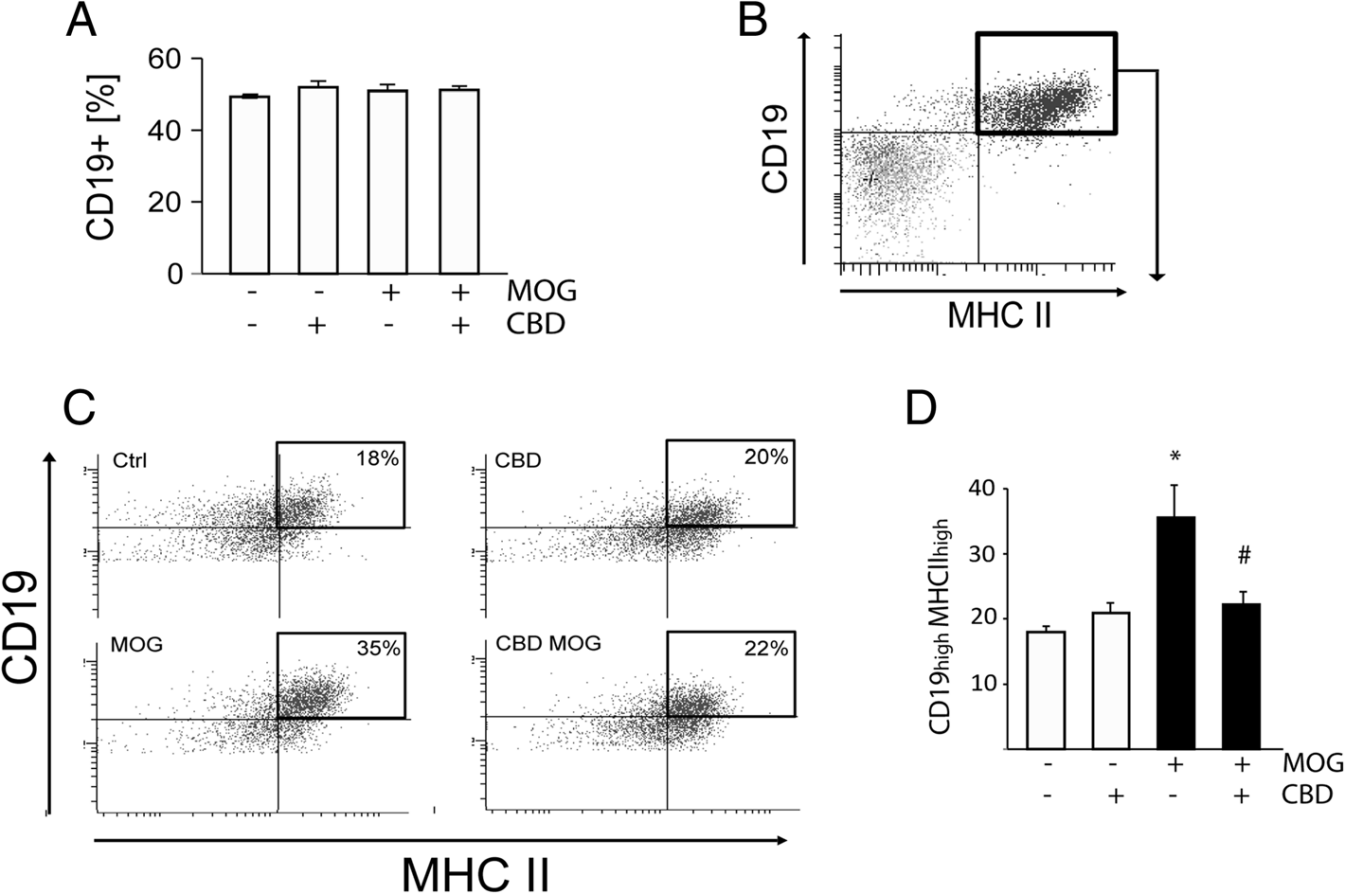
**Upregulation of MHCII in CD19** ^**high**^ **B cells in MOG35-55-stimulated APC/T**_**MOG**_ **co-cultures is decreased by CBD.** APC/T_MOG_ co-cultures were stimulated with MOG35-55, treated with CBD and the percentage of CD19^high^MHCII^high^ B cells determined using flow cytometry. **(A)** Neither treatment affected the number of CD19^+^ B cells; **(B)** dot plot showing representative flow cytometry gating of CD19^+^ B cells expressing MHCII; **(C)** representative dot plot graph of CD19^high^MHCII^high^ cells; **(D)** bar graph showing the mean ± SEM percentage of CD19^high^MHCII^high^ B cells within the total CD19^+^MHCII^+^ cells (taken as 100%). ANOVA *F*(3,8) = 7.6, *P* < 0.05. *P < 0.01 *vs* control, ^#^ *P* < 0.01 *vs* MOG-stimulated cells. *n* = 3.

LAG3 increases were found to reduce APC functions, including antigen presentation by MHCII molecules [12]. Indeed, MHCII expression is an important parameter in determining the antigen presenting efficiency of cells, including B cells, the main peripheral APC. We have, therefore, examined if the CBD-induced increase in LAG3 is accompanied by changes in CD19^+^ B cell APC activities. We found that neither MOG35-55 nor CBD treatments affected the total number of CD19^+^ B cells in APC/T_MOG_ co-cultures (Figure 5A). CD19^high^ B cells were observed to be upregulated in autoimmune pathologies and proposed to be targeted in therapeutic approaches [38]. Indeed, we observed that 18 h MOG35-55 stimulation of APC/T_MOG_ co-cultures resulted in a significant increase in the number of CD19^high^MHCII^high^ cells (35.5% ± 5.0% of all CD19^+^MHCII^+^ cells), reaching almost twice the level observed in control, non-stimulated cells (18.0% ± 0.8%; Figure 5B,C,D). Co-incubation of MOG35-55-stimulated APC/T_MOG_ cells with 5 μM of CBD resulted in a large decrease of CD19^high^MHCII^high^ cells back to the basal level of 22.0% ± 2.0% (*P* < 0.05). CBD did not affect the number of CD19^high^MHCII^high^ cells in non-stimulated, control APC/T_MOG_ co-cultures (20.9% ± 1.5%; Figure 5D).

**Figure 6:**
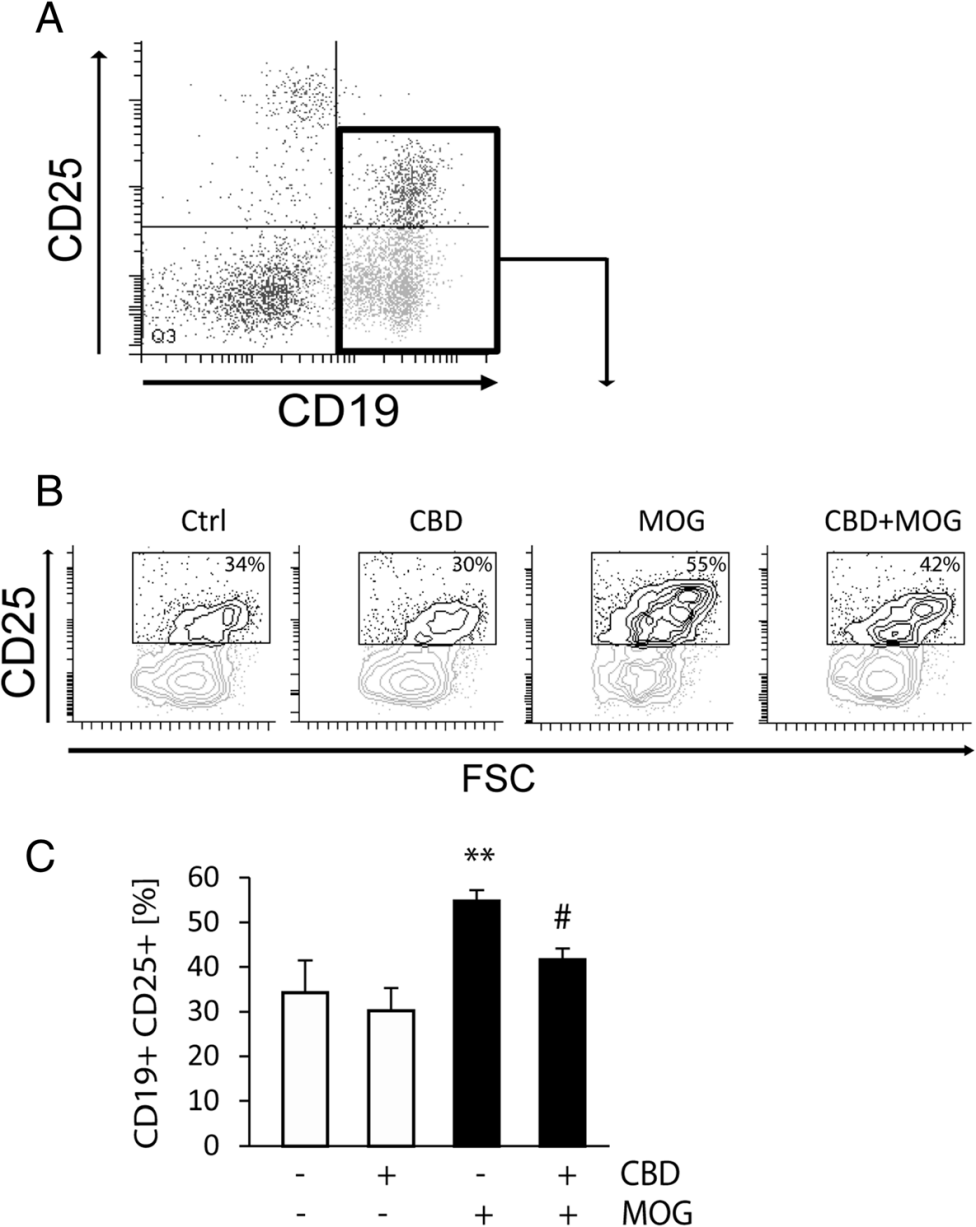
**Upregulation of CD19** ^**+**^ **CD25** ^**+**^ **B cells in MOG35-55-stimulated APC/T**_**MOG**_ **co-cultures is decreased by CBD.** APC/T_MOG_ co-cultures were treated as described above and cells were stained and analyzed by flow cytometry. **(A)** Dot plot showing gating for CD19^+^CD25^+^ cells; **(B)** density gradient graphs for CD25 expression on CD19^+^ cells; **(C)** bar graph showing the mean percent ± SEM of CD19^+^ B cells expressing CD25. ANOVA *F*(3,8) = 8.8, *P* < 0.01 with 100% representing total CD19^+^ cells. **P < 0.01 *vs* non-stimulated cells, ^#^ *P* < 0.01 *vs* MOG35-55-stimulated cells. *n* = 3.

CD25 antigen (that is, IL2 receptor α) is recognized as a marker of CD19^+^ B cells autoimmune activity and is involved in cytokine secretion [39]. We observed that the frequency of CD19^+^CD25^+^ cells was as low as 2.5% ± 0.2% of the total CD19^+^ B cell population in resting naïve spleen-derived APC cells cultured without encephalitogenic T_MOG_ (Table 2). However, the CD19^+^CD25^+^ frequency increased to 34.3% ± 7.2% following co-culturing of splenocytes with T_MOG_ cells (Figure 6B,C). CBD did not affect the number of CD19^+^CD25^+^ cells in non-stimulated APC/T_MOG_ co-cultures (30.2% ± 5.1%). On the other hand, MOG35-55 stimulation of APC/T_MOG_ co-cultures resulted in a significant increase in CD19^+^CD25^+^ cells (up to 54.8% ± 2.4% of the total CD19^+^ cells, *P* < 0.01), and this number was decreased in the presence of CBD to 41.6% ± 2.5% (*P* < 0.05 *vs* MOG35-55-stimulated cells) (Figure 6B,C).

**Figure 7:**
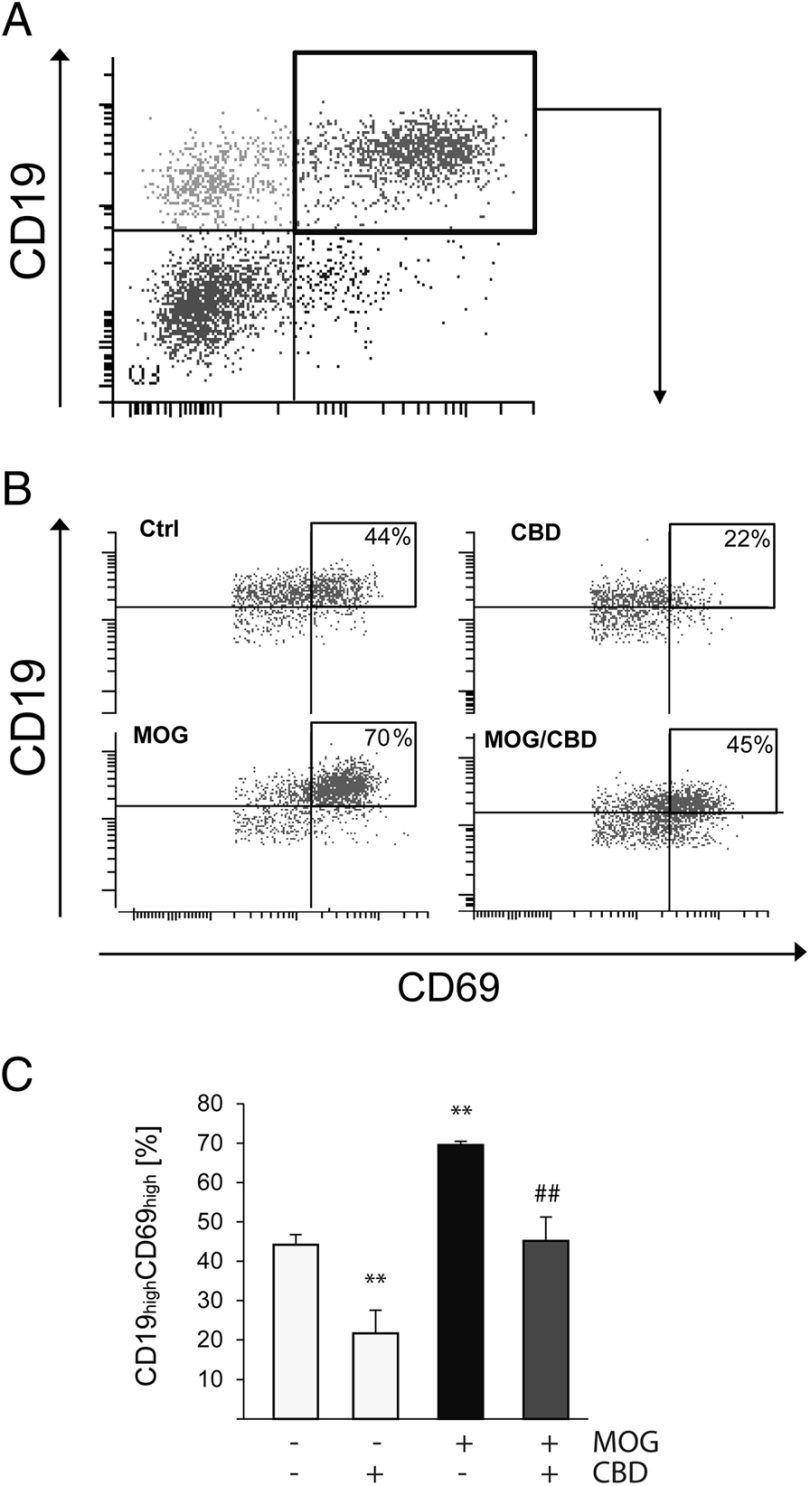
**MOG35-55 increases and CBD decreases the number of CD19** ^**+**^ **CD69** ^**+**^ **B cells in APC/T**_**MOG**_ **co-cultures.** APC/T_MOG_ co-cultures were examined for CD19^+^CD69^+^ cells using flow cytometry. **(A)** Dot plot graph showing gating for CD19^+^CD69^+^ cells; **(B)** representative dot plots showing flow cytometry analysis of CD69 expression on CD19^+^ B cells with a quadrant marking CD19^high^CD69^high^ cells; **(C)** bar graph showing the mean percentages ± SEM of CD19^high^CD69^high^ within CD19^+^CD69^+^ parent population (equivalent to 100%). ANOVA *F*(3,8) = 19.3, *P* < 0.001. **P < 0.01 *vs* control, ^##^ *P* < 0.01 *vs* MOG-stimulated cells. *n* = 3.

CD69 level on CD19^+^ B cells serves as an indication of B cells pro-inflammatory activity. In spleen-derived APC cultured without T_MOG_ cells (resting splenocytes), 13.6% ± 3.4% of the CD19^+^ B cells are CD69 positive and this expression is not significantly affected by CBD treatment (17.4% ± 3.4%; Table 2). However, co-culturing of spleen-derived APC cells with T_MOG_ cells resulted in a remarkable increase in CD69 antigen, particularly on CD19^high^ B cells as this population reached 44.2% ± 2.6% of all CD19^+^CD69^+^ cells (Figure 7B,C). CBD co-incubation decreased the number of CD19^high^CD69^high^ in non-stimulated APC/T_MOG_ co-cultures down to 21.6% ± 5.9% (*P* < 0.01). MOG35-55 stimulation of APC/T co-cultures resulted in a further significant increase in CD19^high^CD69^high^ frequency reaching 70.0% ± 1.0% of the total number of CD19^+^ cells (*P* < 0.01). This increase was reduced in the presence of CBD to 45.2% ± 6.9% - a reduction similar to that observed in non-stimulated APC/T_MOG_ co-cultures (Figure 7B,C).

### LAG3 expression on CD19^+^ B cells is not affected by the presence of CBD

Although LAG3 serves as a CD4 negative co-receptor and thus is mainly expressed on CD4^+^ T cells, its increased levels have been recently reported on B cells as well [40]. In our hands, the basal frequency of CD19^+^LAG3^+^ B cells in APC/T_MOG_ co-cultures was only 6.9% ± 0.4% and was not affected by the presence of CBD (7.9% ± 0.9%; Table 4). Moreover, neither MOG35-55 stimulation nor MOG + CBD combination had any effect on CD19^+^LAG3^+^ cell frequencies in our APC/T_MOG_ co-cultures (6.0% ± 0.4% and 5.7% ± 0.9%, respectively). Thus, in contrary to the situation in T cells, LAG3 expression in B cells is not regulated by either MOG35-55 stimulation or CBD treatment.

**Table 4:** **Level of LAG3 expression on CD19** ^**+**^ **B cells in the presence of CBD in APC/T**_**MOG**_ **co-cultures**

**Treatment**	**CD19** ^**+**^ **LAG3** ^**+**^ **(%)**
Control	6.9 ± 0.4
CBD	7.9 ± 0.9
MOG35-55	6.0 ± 0.4
CBD + MOG35-55	5.7 ± 0.9

APC/T_MOG_ co-cultures were stimulated with MOG35-55 and CBD. The level of LAG3 was measured by flow cytometry and is expressed as mean ± SEM% of CD19^+^ B cells positive for LAG3. ANOVA *F*(3,8) = 1.8, *P* > 0.05 (*n* = 3).

## Discussion

**Scheme 1:**
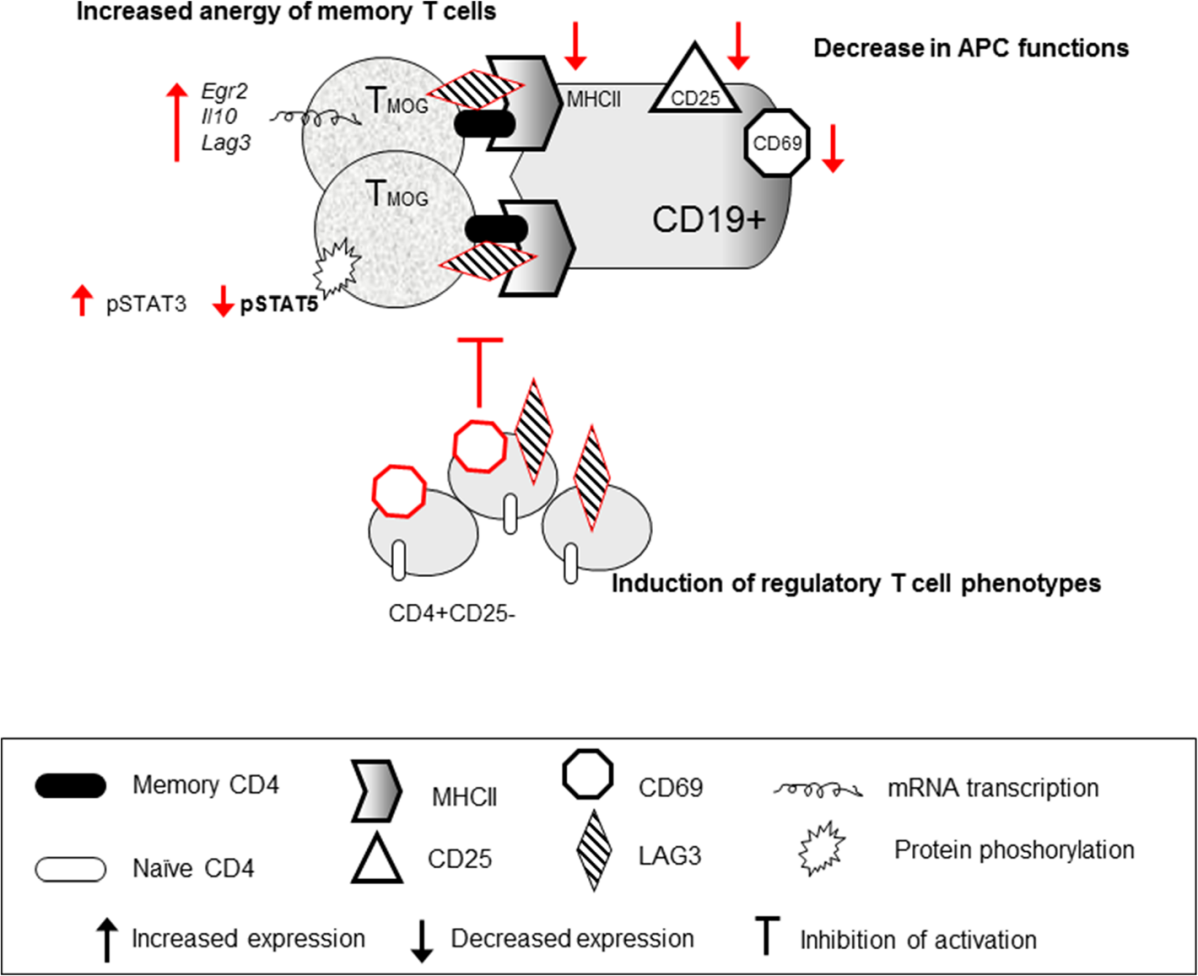
**Schematic diagram showing the main effects of CBD (in red) in MOG35-55-stimulated APC/T**_**MOG**_ **co-cultures.** The symbols used in the scheme are explained below.

In this work, we studied the immunoregulatory effects of CBD using an *in vitro* model of T_MOG_ cells co-cultured with spleen derived APCs and other accessory cells. We found that CBD exerts its immunoregulatory effects by a *de novo* induction of regulatory CD4^+^CD25^−^ T cells that express high levels of suppressive CD69 and LAG3 molecules. This induction was accompanied by an increase in EGR2 transcription, as well as EGR2 and IL-10-dependent anergy of stimulated memory T_MOG_ cells and in a shift in STAT3/STAT5 activation balance. Moreover, we observed decreased antigen presenting capabilities (indicated by lower MHCII expression) and decreased pro-inflammatory activity (indicated by lower CD69 and CD25 expression) of MOG35-55-stimulated B cells in the presence of CBD (Scheme 1).

Naturally occurring CD4^+^CD25^+^ regulatory T cells (nTreg) play an indispensable role in preventing autoimmunity mostly *via* Foxp3-dependent transcription [41]. Reduced frequency and reduced suppressive functions of nTreg (for example, *via* impaired STAT5 activity) have been observed in MS patients [42,43]. This phenomenon was mimicked in our *in vitro* autoimmune model in which MOG35-55 stimulation resulted in reduced number of CD4^+^CD25^+^ nTreg cells, demonstrating the suitability of this system for studying autoimmune inflammation and for identifying possible treatments. Interestingly, CBD did not reverse the MOG35-55-induced reduction in CD4^+^CD25^+^ nTreg cells. Thus, we assume that other regulatory mechanisms mediate the anti-inflammatory activity of CBD in this system and in Th17-driven EAE.

Non-classical CD4^+^ T regulatory cells are induced in the periphery and have a pivotal role in maintaining immune tolerance, mainly *via* Foxp3-independent mechanisms. CD4^+^CD25^−^ T cells have been reported to be the main source of inducible regulatory phenotypes [23,24]. These cells exert their regulatory functions *via* a number of unique surface regulatory molecules, including LAG3 and CD69, acting separately or synergistically to diminish inflammation, including Th17-driven autoimmunity [19,44]. Such immunoregulation involves impaired antigen stimulation of T memory cells, impaired interactions of memory T cells with APC, promoting negative shift in Teff/Treg balance and/or inducing anergy in activated T cells. Our results show that CBD treatment upregulates the levels of CD69 as well as of LAG3 molecules on CD4^+^CD25^−^ T cells (splenocytes) co-cultured with T_MOG_ cells. Moreover, in MOG35-55-stimulated APC/T_MOG_ co-cultures, CBD effect was augmented and led to the induction of regulatory phenotypes double positive for CD69 and LAG3, suggesting boosted immunoregulation upon self-antigen activation.

CD69 was shown to serve as a constitutive brake for Th17 differentiation [18,19]. CD69 has a negligible expression in resting T and B lymphocytes but is rapidly induced on these cells upon their activation [45]. CD69 was initially considered as an early activation marker and its key role in inducing regulatory mechanisms is well documented [18,46,47]. Although CD69 deficiency does not affect basal lymphocyte function, it leads to augmented autoimmunity including murine collagen-induced arthritis, lupus, or autoimmune myocardiopathy ([48]; see [18] for additional refs). Indeed, CD69-deficient naïve T cells preferentially differentiate toward Th17, secrete high amounts of IL-17 following antigen stimulation *in vitro* and *in vivo* that is accompanied by increased STAT3/RORγt and diminished STAT5 activities [19]. In agreement with this, CD69 induction was shown to increase STAT5 suppressory activity and to decrease IL-17 release [19]. These observations are in line with our results showing that CBD upregulates CD69 on CD4^+^CD25^−^ T cells and that CBD treatment leads to decreased STAT3 and increased STAT5 phosphorylations in T_MOG_ cells.

LAG3 is similar to CD4 in its structure and genomic organization and serves as a high affinity, negative competitor of CD4 in binding MHCII [49,50]. LAG3 plays a crucial role in immune homeostasis involving antigen-stimulated T cells and has become a golden standard in tracking regulatory T cell phenotypes in many species, including human [44,51]. Interestingly, similarly to the case of CD69, LAG3 deficiency does not result in basal lymphocyte dysfunction but rather impairs tolerogenic mechanisms upon antigen activation leading to exacerbated autoimmunity. Basal levels of LAG3 in quiescent immune cells are low and this level is increased in activated T cells, including Th17 subtype, as a negative feedback loop response [20,49,50,52,53]. LAG3 was shown to reduce antigen-induced T cell expansion, to limit memory T cell pools [13], and to prevent various types of autoimmunity in mice [52,54-56]. LAG3 increased expression on regulatory cells has been accompanied by decreased IL-17 and increased IL-10 secretions [24,51,57]. Thus, the increase of LAG3 by CBD treatment reported here is in agreement with our previous results showing that CBD lowers IL-17 expression and secretion [9].

Increased LAG3 and IL-10 expressions are the most reliable markers of ongoing EGR2-driven anergic processes in activated T cells [24,25,57-59]. Forced expression of EGR2 in naïve T cells converts them into LAG3 expressing and IL-10 secreting regulatory cells [25] while EGR2 deficiency results in lupus-like autoimmunity [60], as well as increased STAT3 phosphorylation and IL-17 secretion [34]. Indeed, here we show that CBD treatment, that, as discussed above, decreases IL-17 secretion [9] promotes expression of EGR2 in T_MOG_ cells along with enhanced expression of LAG3 and IL-10 as well as of STAT5 phosphorylation.

Anergy-related decrease in T cell division and cytokine secretion (exhaustion) is driven by a well-defined set of tolerogenic genes and inhibitory proteins activated in response to antigen stimulation and is increased *via* EGR2 activation [16,61,62]. Accordingly, our gene transcript analysis of the effect of CBD on stimulated T_MOG_ cells reveals enhanced anergy-related transcription profile. A number of transcripts are known to execute cell cycle arrest and EGR2-dependent tolerogenic events [16,62-64]. Several of these transcripts were found by us to be upregulated when CBD was added together with MOG35-55, including NFATc1, Casp4, Ndrg1, Lag3, and Icos. In addition, Akt activation, known for its role in restraining/reversing anergy [27,65] was upregulated by MOG35-55-activation and reduced in the presence of CBD, adding to the anergy-facilitating environment induced by this cannabinoid.

MHCII-dependent APC function of B cells is required for the induction of MOG35-55-induced Th17 differentiation, memory T cells’ expansion and EAE development [66]. B cells are the main source of IL-6 cytokine that controls Th17 differentiation [11]. Indeed, deficiency in IL-6-secreting B cells prevents IL-17 production and EAE [67]. This corresponds well with our recent observations that CBD decreases IL-6 and IL-17 release from stimulated T_MOG_ cells as well as lowers EAE severity [8,9]. Increased LAG3 was shown to impair antigen presenting functions of immune cells [68]. Thus, CBD-induced increase in LAG3 levels could lead to the reduced antigen presenting capacities in B cells as indeed indicated by lower MHCII levels in our system, along with the reduction in their pro-inflammatory activity as indicated by the lower levels of CD25 and CD69, molecules that serve as signature markers for B cell autoimmune activation [39,69].

Interestingly, the CBD-induced shift in STAT3/STAT5 phosphorylation ratio, the decrease in Akt activity in T_MOG_ cells as well as the increases in CD69 and LAG3 expression on accessory T cells are also observed in APC/T_MOG_ co-cultures in the absence of MOG35-55 stimulation. This suggests that CBD may be raising an inherent basal activation threshold in these encephalitogenic T cells. Clearly, CBD-induced immunosuppression is amplified under activation conditions as evidenced by the induction of CD4^+^CD25^−^CD69^+^LAG3^+^ cell population in CBD-treated MOG35-55-stimulated APC/T_MOG_ co-cultures, accompanied by EGR2-dependent anergy in T_MOG_ cells co-treated with CBD and MOG35-55. Moreover, by changing the balance of STAT3/STAT5 transcription factors and by increasing IL-10 expression in stimulated T_MOG_ cells, CBD seems to turn pathogenic Th17 into non-pathogenic or even anti-inflammatory T cells. This phenomenon is known as a STAT5-dependent cytokine switch of memory T cells and has a self-regulatory function [70,71]. It may involve inhibition of neighboring cells, such as APC [72].

## Conclusions

Cannabinoid regulation of immune cell function was studied and reviewed by several other groups [1,2,73,74]. Various cannabinoids have been shown to decrease maturation, proliferation, migration, adhesion, and cytokine secretion from activated immune cells. Our group and others identified MAPKs, AP-1, NFkB, STAT, and NFAT pathways as well as ROS formation to be targeted by cannabinoids in activated immune cells [1,2,30,73,74]. However, most of the data addressing these pathways was obtained with the use of naïve immune cells activated by non-specific activators, like phytohaemaglutinin, PMA/Ionomycin, and/or bacterial endotoxins, that only to a limited extent mimic the *in vivo* inflammation, particularly of autoimmune background. Moreover, although cell-mediated immunoregulation has been proved to be the most effective way of restoring immune homeostasis, the effect of cannabinoids on cell-mediated immunoregulation has been barely investigated so far. Recent work of Hegde *et al*. [75] showed that myeloid-derived suppressor cells may mediate anti-inflammatory effects of CBD in autoimmune hepatitis suggesting that cannabinoids indeed have a potential to involve regulatory cells in their anti-inflammatory effects. Here, we are using an *in vitro* model, composed of memory T cells stimulated with myelin antigen in the presence of peripheral accessory cells to study the complex multicellular interactions driving the autoimmune inflammation and to study the effects of CBD on these processes. This approach mimics much better the *in vivo* autoimmune processes that take place in EAE mice. Our *in vitro* system allowed us to show that CBD anti-inflammatory activities in autoimmune conditions involve *de novo* induction of regulatory phenotypes, transcriptional and functional reprogramming of memory T cells toward non-pathogenic and even inhibitory cells (increased IL-10), as well as reduced activation of B cells, the main antigen presenting cells in the periphery.

## Abbreviations

APC: antigen presenting cells
CBD: cannabidiol
CD: cluster of differentiation
EAE: experimental autoimmune encephalomyelitis
EGR2: early growth response 2
LAG3: lymphocyte-activation gene 3
MHCII: major histocompatibility complex class II
MOG35-55: myelin oligodendrocyte glycoprotein 35 to 55
STAT: signal transducer and activator of transcription
T_MOG_: MOG35-55-specific T cells
